# All Tangled Up: Meter-Long Gastric Trichobezoar Causing Multiple Intussuscepting Points in an Adult

**DOI:** 10.7759/cureus.36651

**Published:** 2023-03-24

**Authors:** Daniel Jia Wei Lee, Kang Ler Fong, Yeow Chun Tee, Oscar B Aldridge

**Affiliations:** 1 General Surgery, Fiona Stanley Hospital, Perth, AUS

**Keywords:** trichophagia, gastroscopy, gastrotomy, intussusception, trichobezoar

## Abstract

Gastric trichobezoars are a result of trichophagia secondary to trichotillomania, and can result in severe complications such as perforation or intussusception. We present the case of a 19-year-old female with multiple points of intussusception secondary to a large gastric/small intestine trichobezoar and describe our approach to diagnosis and eventual removal of the bezoar.

## Introduction

Bezoars are rare conglomerations of usually indigestible materials within the gastrointestinal tract. These materials can be from a variety of sources, including hair (trichobezoar), insoluble plant material (phytobezoar), medications (pharmacobezoar), milk proteins (lactobezoar) or foreign bodies [1]. Risk factors for the development of gastric bezoars can be categorised into medical conditions (such as anorexia nervosa, pica or cystic fibrosis), anatomical abnormalities (such as pyloric stenosis or cholecystogastric/duodenal fistulas) and gastric motility disorders (such as gastroparesis secondary to diabetes or previous gastric surgeries) [2]. Younger (and especially female patients) often present with a pica history, with resultant trichobezoar or foreign bodies as the material source. In contrast, older patients present with gastroparesis as the underlying cause, with phytobezoars or pharmacobezoars as the primary material of the bezoar. Patients with bezoars can potentially be asymptomatic but more often present with epigastric abdominal pain, nausea, vomiting, dyspepsia or weight loss [3]. Management depends on the underlying cause of the bezoar, such as chemical dissolution, prokinetics or endoscopic/surgical removal [4-6].

Whilst rare, complications requiring surgical intervention can arise from the presence of a bezoar, such as gastric ulceration/perforation with resultant peritonitis, bowel obstruction or intussusception [7,8]. Especially within the adult population, intussusception is a particularly rare pathology (0.003%-0.02% of adult hospitalisations) of which around 90% of cases demonstrate a pathological lead point, such as hamartomatous polyps secondary to Peutz-Jeghers syndrome, Meckel’s diverticulum or adenocarcinoma [9].

We report a case of adult intussusception with multiple points of intussusception secondary to trichobezoar, and explore the diagnostic and management process.

## Case presentation

**Figure 1:**
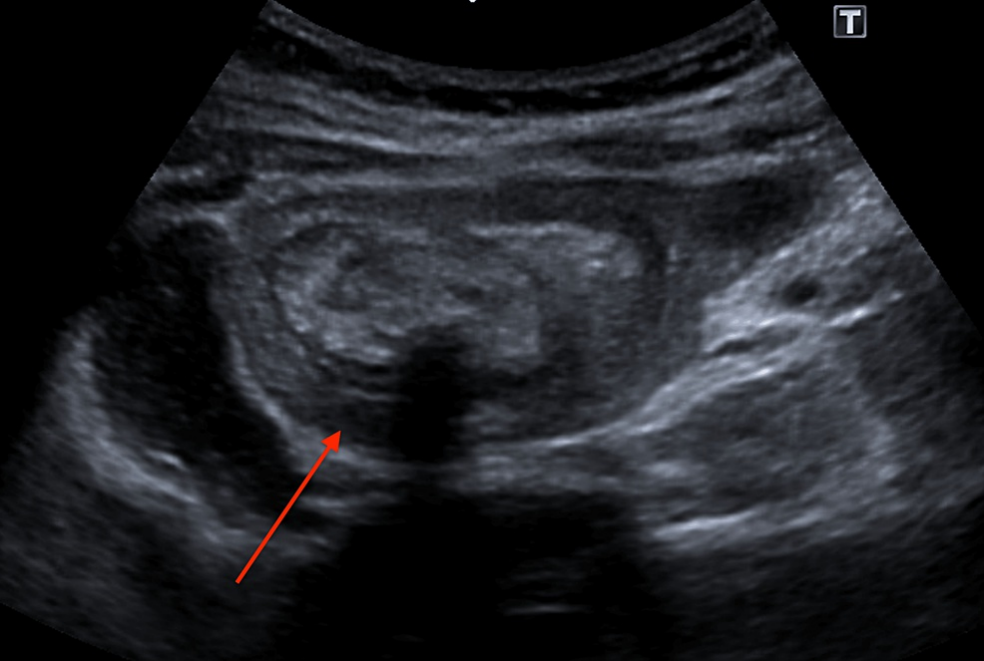
Ultrasound scan six years prior (aged 13) demonstrating target sign characteristic of intussusception

A 19-year-old female was transferred from a peripheral hospital to our tertiary centre for ongoing management of multiple intussuscepting bowel loops (gastro-duodenal, jeju-jejunal) demonstrated on computerised tomography (CT) of her abdomen. She had presented with a four-month history of increasing generalised abdominal pain with associated nausea and diarrhoea, with intermittent flares of severe pain which precipitated her presentation to hospital. Of note in her background was a resection of osteochondroma from medial thigh at age 14, a previous laparotomy with removal of gastric trichobezoar aged 13 (intraoperatively diagnosed during laparotomy from ultrasound at the time suggesting intussusception (Figure 1), and ongoing well-controlled anxiety. She denied eating hair in the recent period (although stated may have been doing so unconsciously).

**Figure 2:**
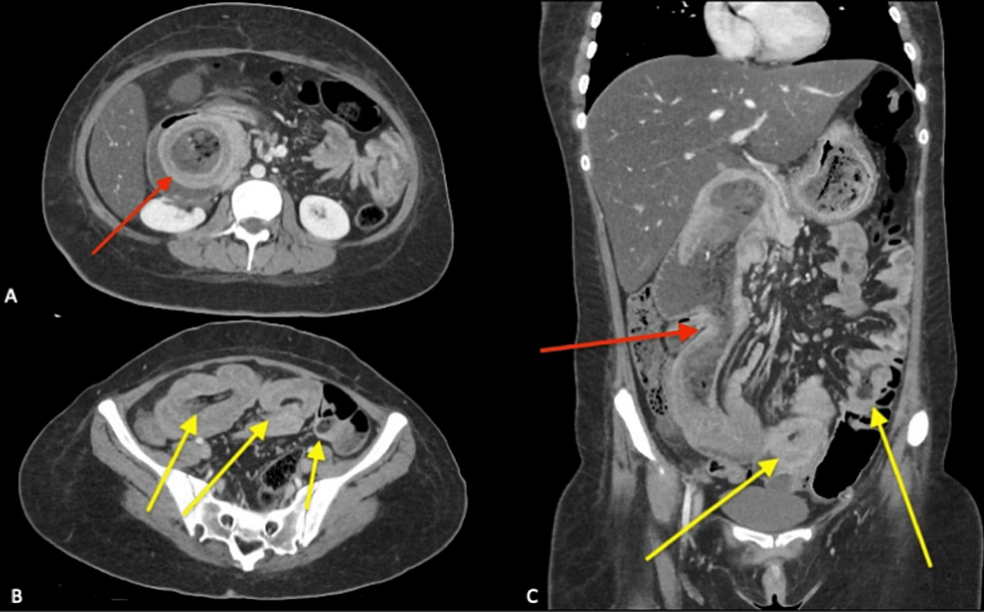
CT scan demonstrating multiple intussuscepting points. Red: Gastroduodenal intussusception. Yellow: Multiple separate jejuno-jejunal intussusception points

On examination, her vitals were within normal limits and she was afebrile. Her abdomen had fullness to palpation in the epigastric and right hypochondrial regions with associated tenderness but no peritonism. Her blood tests demonstrated normal electrolytes and inflammatory markers, haemoglobin levels, renal and liver function. Her CT scan demonstrated multiple intussuscepting bowel loops, with gastro-duodenal intussusception seen along with multiple jejuno-jejunal segments (Figures 2A-2C).

**Figure 3:**
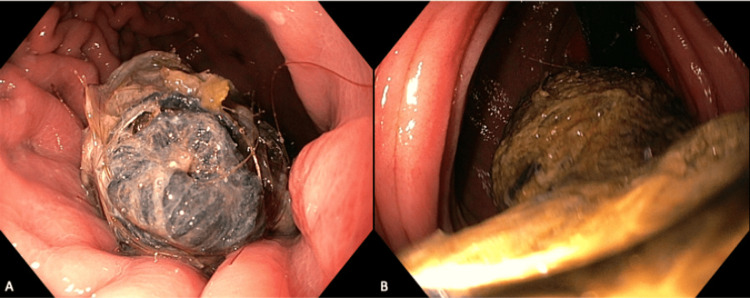
Gastroscopy views. A: Head of trichobezoar in stomach. B: Most distal view of gastroscope demonstrating continuation of tail of trichobezoar into jejunum

**Figure 4:**
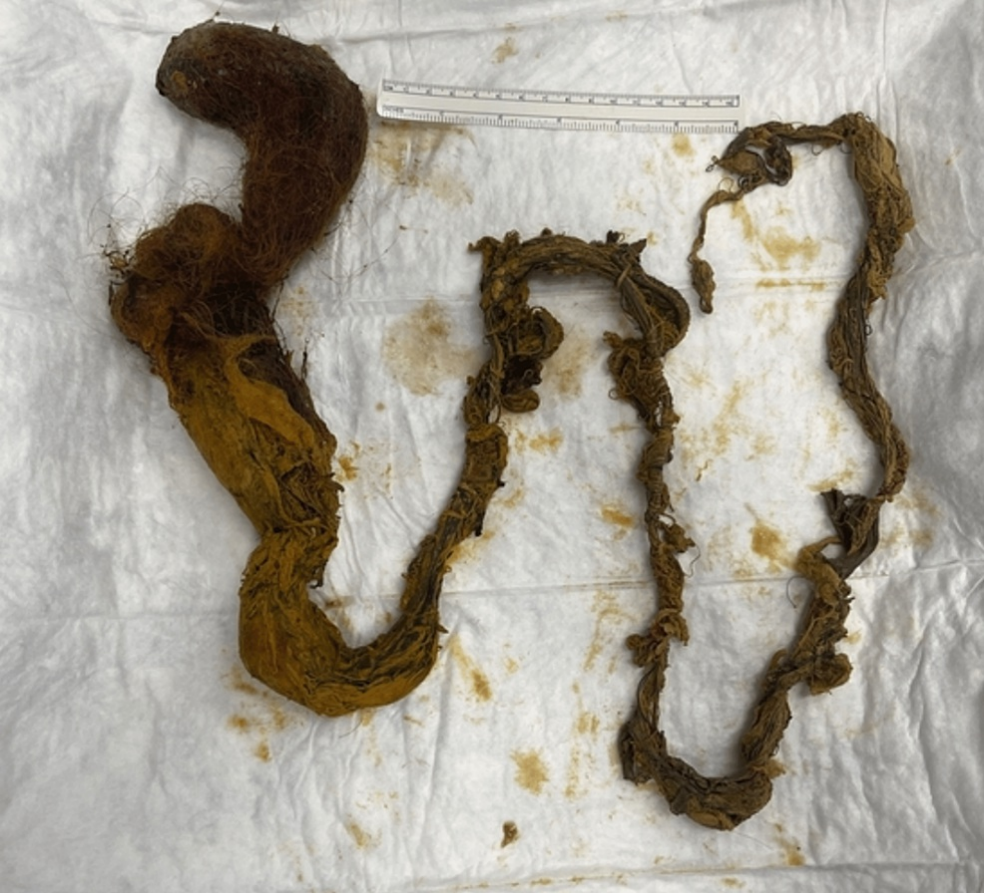
Completely intact removal of trichobezoar measuring 1m

Given her age and the multiple intussuscepting points, there was a need to rule out a potential intramural cause of the intussusception such as polyp disease. As such, she first underwent a gastroscopy as a preliminary investigation (Figures 3A, 3B), demonstrating a large trichobezoar extending past the limit of the gastroscope. There was no evidence of gastro-duodenal intussusception at the time. She proceeded to a laparoscopic-assisted gastrotomy through a 5 cm midline incision to remove the trichobezoar. Her stomach was delivered to the midline wound and an Alexis retractor was utilised to prevent wound contamination. A trichobezoar measuring 1m in length was retrieved (Figure 4) and completion endoscopy through the gastrotomy demonstrated complete removal of the trichobezoar, healthy mucosa of the stomach and duodenum/proximal jejunum, and no intussuscepting bowel loops remaining. This was confirmed on a review of the bowel laparoscopically after closure of the gastrotomy and the midline fascial defect.

Post-operatively she progressed well with no complications, with improvement in pain and was discharged home on a soft diet three days after her operation. She was reviewed by the Psychiatry team during her admission with plans for outpatient community mental health support after her discharge.

## Discussion

The pulling of hair (trichotillomania) and subsequent ingestion of hair (trichophagia) is a neuropsychological disorder that is usually a response to a stress-inducing event or boredom, reported more commonly in females (9:1 ratio). After the pulling of hair, some patients would inspect or eat the hair (trichophagia), and when this occurs the patient can be at risk of trichobezoar formation. It is important to note that this process may happen spontaneously, being performed while the patient is unaware or unfocused on the action, such as in our patient [10]. The resultant trichobezoar could then proceed to cause further symptoms of abdominal pain, nausea, vomiting and obstipation as it progressively causes further obstruction of the gastrointestinal tract. It can also influence nutritional intake, with failure to thrive, anaemia and hypoalbuminaemia occurring as a result of protein wasting and chronic gastritis secondary to the constant gastric irritation from the trichobezoar [11].

The pathophysiology of trichobezoar development relates to the structure of hair itself, with its smooth surface and resistance to digestion resulting in it being matted into a ball within the gastric mucosa folds. This eventually becomes large enough to cause gastric distension and delay stomach emptying as more hair gets ingested [11]. A further concern from the development of the gastric trichobezoar is the resulting tail that develops from the main gastric trichobezoar, known as Rapunzel syndrome. This can cause downstream intestinal obstruction, with single-point intussusception being rarely reported in the literature. In addition, most intussusception cases are reported in children, in contrast to our adult patient with multiple intussusception points within the small intestine [12]. As such, when investigating an adult patient who presents with multiple intussuscepting points within the gastrointestinal tract, one needs to consider familial syndromes that predispose to polyp formation such as Peutz-Jegher syndrome, familial juvenile polyposis syndrome and Cowden syndrome [9].

The mainstay of treatment for trichobezoars is operative intervention. Most non-operative interventions that may be suitable for other types of bezoars such as chemical or enzymatic dissolution are unlikely to work in the trichobezoar given its physical composition. Considerations for operative intervention include endoscopic retrieval of the trichobezoar, laparoscopic removal, and laparotomy. Endoscopic management is usually only successful in very small gastric trichobezoars, where they can be removed intact. Larger bezoars may be fragmented down but there is a risk of these fragments migrating down into the distal gastrointestinal tract and causing luminal obstruction. Furthermore, if multiple passes with the gastroscope are required for the retrieval of the trichobezoar fragments, the risk of trauma, pressure ulceration, oesophagitis and oesophageal perforation increases. Performing removal of trichobezoars laparoscopically can result in better cosmesis, less post-operative complications and admission periods. However, there remains the risk of contamination secondary to hair spillage during the retrieval process, and the difficulty of examining the rest of the intestine to ensure no satellite trichobezoars remain makes laparoscopic removal less appealing. Laparotomy has been reported as 100% successful in the literature for removal of trichobezoars, with the risk of complications such as skin infection and post-operative ileus being low. It also has the added benefit of easy access to the rest of the intestine to assess for satellite trichobezoars [12].

Our approach in the management of this patient combines the strengths of both the laparoscopic and open approach to removal of this large trichobezoar. This resulted in a smaller incision to retrieve the large trichobezoar, minimal risk of wound contamination, faster return to bowel function and shorter hospitalisation in the post-operative period. This case highlights the importance of careful consideration and tailoring of the investigative and management phase based on patient factors, to achieve optimal and holistic patient care.

## Conclusions

Gastric trichobezoars can present in an indolent fashion, and careful consideration into specific risk factors is required in its investigative workup, especially when it presents with complications such as intussusception or bowel perforation. A broader differential diagnosis needs to be evaluated particularly in the adult patient who presents with intussusception to ensure more malignant conditions are not missed. Lastly, a laparoscopic-assisted gastrotomy can be a safe and effective management strategy in removing large gastric trichobezoars with the added benefits of smaller wounds with reduced risk of infection and a faster return to function.
